# Association between nirsevimab coverage and pediatric emergency department visits for bronchiolitis in 12 European countries: An interrupted time-series analysis

**DOI:** 10.1371/journal.pmed.1005223

**Published:** 2026-09-03

**Authors:** Léa Lenglart, Luigi Titomanlio, Ilaria Alberti, Laura Almeida, Camille Aupiais, Esra Akyüz Özkan, Michael Barrett, Romain Basmaci, Ahmet Birbilen, Dorine Borensztajn, Catarina Braz Schönenberger, Silvia Bressan, Danilo Buonsenso, Teresa Campos, Susana Castanhinha, Antonio Chiaretti, Gemma Claret, Francesca De Zan, Sheena Durnin, Laszlo Fodor, Borja Gomez, Susanne Greber-Platzer, Romain Guedj, Alexander Huege, Fiona Leonard, Ian K. Maconochie, Robin Marlow, Gregorio Milani, Miguel Angel Molina Gutierrez, Anna Maria Musolino, Rianne Oostenbrink, Zanda Pucuka, Simon Rivière, Damian Roland, Malin Ryd Rinder, Maria Chiara Supino, Ozlem Teksam, Victoria Trenchs, Zaba Valtuille, Bento Vanda, Sanne Vrijlandt, Naim Ouldali, Ruud G. Nijman, the EPISODES Study Group

**Affiliations:** 1 Pediatric Emergency Department, Robert Debré University Hospital, Assistance Publique–Hôpitaux de Paris, Paris Cité University, Paris, France; 2 Université Paris Cité and Université Sorbonne Paris Nord, Inserm, IAME, Paris, France; 3 Pediatric Emergency Department, Fondazione IRCCS Ca’ Granda Ospedale Maggiore Policlinico, Milan, Italy; 4 Department of Clinical Science and Community Health, University of Milan, Milan, Italy; 5 Centro Hospitalar e Universitário de São João, University of Porto, Porto, Portugal; 6 Pediatric Emergency Department, Jean Verdier Hospital, Bondy, Assistance Publique-Hôpitaux de Paris, Sorbonne Francecité, France; 7 INSERM UMR 1123, ECEVE, Paris, France; 8 Pediatric Emergency Department, Ondokuz Mayıs University, Samsun, Turkey; 9 Pediatric Emergency Department, Children’s Health Ireland at Crumlin, Dublin, Ireland; 10 Women’s and Children’s Health, University College Dublin, Dublin, Ireland; 11 Pediatric Emergency Department, Louis Mourier Hospital, Colombes, Assistance Publique–Hôpitaux de Paris, Paris Cité University, Paris, France; 12 Division of Pediatric Emergency Medicine, Department of Pediatrics, Hacettepe University School of Medicine, Ankara, Turkey; 13 Department of Pediatrics, NoordWest Ziekenhuisgroep, Alkmaar, The Netherlands; 14 Departamento da Criança e do Jovem- Urgencia Pediatrica, Hospital Prof. Doutor Fernando da Fonseca, Amadora, Portugal; 15 Division of Pediatric Emergency Medicine, Department of Women’s and Children’s Health, University Hospital of Padova, Padova, Italy; 16 Department of Woman and Child Health and Public Health, Fondazione Policlinico Universitario Agostino Gemelli IRCCS, Rome, Italy; 17 Area Pediatrica, Dipartimento di Scienze della Vita e di Sanità Pubblica, Università Cattolica del Sacro Cuore, Rome, Italy; 18 Pulmonology Unit, Department of Pediatrics, Hospital Dona Estefania, Centro Hospitalar Lisboa Central, Lisbon, Portugal; 19 Pediatric Emergency Department, Hospital Joan de Déu Barcelona, University of Barcelona, Barcelona, Spain; 20 Environment Effects on Child/Adolescent Well-being, Institut de Recerca Sant Joan de Déu, Barcelona, Spain; 21 Department of Pediatric Emergency Medicine, Children’s Health Ireland at Tallaght, Dublin, Ireland; 22 Discipline of Paediatric Medicine, Trinity College Dublin, Dublin, Ireland; 23 Pediatric Emergency Department, Szent Gyorgy University Teaching Hospital of Fejer County, Szekesfehervar, Hungary; 24 Paediatric Emergency Department, Biobizkaia Health Research Institute, Hospital Universitario Cruces, Barakaldo, Basque Country, Spain; 25 Pediatric Emergency Unit, Clinical Division of Pediatric Pulmonology, Allergology and Endocrinology, Department of Pediatrics and Adolescent Medicine, Medical University of Vienna, Vienna, Austria; 26 Pediatric Emergency Department, Armand Trousseau Hospital, Assistance Publique–Hôpitaux de Paris, Sorbonne Université, Paris, France; 27 CRESS Inserm U-1153 Paris, Epopé Team, Paris, France; 28 Department of Pediatric Emergency Medicine, Division of Medicine, St. Mary’s Hospital, London, United Kingdom; 29 Imperial College NHS Healthcare Trust, London, United Kingdom; 30 Center for Pediatrics and Child Health, Faculty of Medicine, Imperial College London, London, United Kingdom; 31 Emergency Department, Bristol Royal Hospital for Children, Bristol, United Kingdom; 32 Faculty of Health and Applied Sciences, University of the West of England, Bristol, United Kingdom; 33 Pediatric Emergency Department, Hospital Universitario La Paz, Madrid, Spain; 34 Emergency Department, Bambino Gesù Children’s Hospital, IRCCS, Rome, Italy; 35 Department General Pediatrics, ErasmusMC – Sophia, Rotterdam, The Netherlands; 36 Paediatric Emergency Department, Children’s Clinical University Hospital, Riga Stradins University, Riga, Latvia; 37 SAPPHIRE Group, Population Health Sciences, Leicester University, Leicester, United Kingdom; 38 Pediatric Emergency Medicine Leicester Academic (PEMLA) Group, Leicester Hospitals, Leicester, United Kingdom; 39 Pediatric Emergency Department, Astrid Lindgrens Children’s Hospital, Karolinska University Hospital, Stockholm, Sweden; 40 Department of General Pediatrics, Pediatric Infectious Disease and Internal Medicine, Robert Debré University Hospital, Assistance Publique-Hôpitaux de Paris, Paris, France; 41 Paris Cité University, Paris, France; 42 Section of Paediatric Infectious Diseases, Department of Infectious Diseases, Imperial College London, London, United Kingdom; London School of Hygiene and Tropical Medicine, UNITED KINGDOM OF GREAT BRITAIN AND NORTHERN IRELAND

## Abstract

**Background:**

Nirsevimab was introduced to prevent Respiratory Syncytial Virus (RSV) bronchiolitis in infants. Despite its proven effectiveness and similar guidelines across Europe, bronchiolitis reduction varied across Europe. This study explored whether nirsevimab coverage among targeted populations (born in- and out-of-season) was associated to this variability.

**Methods and findings:**

We used routinely collected data from 27 pediatric emergency departments across 12 European countries (01/2018–03/2024). All bronchiolitis cases aged <12 months were included. Regions were classified as intervention or control based on nirsevimab implementation. Coverage was obtained from official regional reports. We estimated changes in monthly case using controlled interrupted time-series models accounting for seasonality and temporal trends, by age-groups (0–3 and 3–12 months) and regions during the *intervention* period (10/2023–03/2024) and examined their correlation to coverage using Pearson’s test. Urinary tract infections served as a control outcome.

We included 107,088 bronchiolitis cases. Reductions varied widely across regions and age-groups, from +0.6% (95% CI [−9.4, +20.5]) to −61.0% (95% CI [−67.8, −35.2]). Coverage strongly correlated with bronchiolitis reduction across age-groups and regions (*ρ* = −0.86, *p* = 0.001). Bronchiolitis trends in *control* countries remained stable, as did urinary tract infections. As an ecological study, causality cannot be inferred and unmeasured regional differences may have contributed to the variability.

**Conclusions:**

This multinational interrupted time-series analysis identified coverage as a key factor associated to bronchiolitis reduction across age-groups and European regions. Implementation choices across Europe likely influenced coverage, highlighting that nirsevimab benefits depend not only on its intrinsic efficacy, but also on how effectively it is delivered.

## Author summary

**Why was this study done?:**

- Nirsevimab, a new long-lasting monoclonal antibody targeting Respiratory Syncytial Virus (RSV), was introduced across Europe in 2023.
- Although clinical trials and real-life effectiveness studies showed high effectiveness, the reduction in bronchiolitis observed after implementation varied substantially across Europe.
- Understanding why this variability occurred is important to maximize the public health impact of RSV prevention programs.

**What did the researchers do and find?:**

- We analyzed data from 27 pediatric emergency departments within regions implementing nirsevimab or not, to calculate the percentage of change in bronchiolitis cases aged under 12 months after the start of the intervention.
- We collected information on nirsevimab coverage among eligible infants and examined whether coverage was associated with the change in bronchiolitis cases.
- We found marked differences in bronchiolitis reductions across regions and age groups, with higher nirsevimab coverage being strongly associated with larger reductions in cases.

**What do these findings mean?:**

- Regions achieving high coverage were associated with a larger reduction in bronchiolitis visits.
- The public health benefit of nirsevimab depends not only on its biological effectiveness but also on how prevention programs are implemented and delivered.
- As this is an ecological study relying on routinely collected regional data, causality cannot be inferred and we cannot exclude that unmeasured regional differences may partly explain the observed variation in bronchiolitis reduction.

## Abstract 3

Using an interrupted time-series analysis, Léa Lenglart and colleagues explore whether heterogeneity in coverage in European countries of the RSV- targeting monoclonal antibody nirsevimab associates with differences in case counts of bronchiolitis in infants.

## Introduction

Acute bronchiolitis, the most frequent lower respiratory tract infection in infants, places a substantial burden on children’s health. Respiratory Syncytial Virus (RSV) is the leading causative agent of bronchiolitis [1] and is estimated to be responsible for 33.1 million cases of lower respiratory tract infections in children younger than 5 years of age annually worldwide [2]. It is also a leading cause of pediatric emergency department (PEDs) visits globally [3].

The recent introduction of RSV immunization [4,5] is a revolution in the field of RSV prevention in infants. Indeed, in September 2023, nirsevimab was first implemented in 3 European countries: France, Spain and Luxembourg, in the prevention of RSV-bronchiolitis in infants. Since its first implementation, many studies demonstrated its real-world effectiveness in primary care visits [6], PEDs visits [7–9], inpatient admissions [10–12] and intensive care hospitalizations [13,14].

However, despite very similar immunization guidelines, the recent literature reports substantial heterogeneity in the number of bronchiolitis cases averted across regions implementing nirsevimab [15,16]. This variability raises concerns about the overall public health impact of current strategies and, more importantly, about the factors driving these differences. Since RSV testing is not systematically performed in routine PED care across Europe, overall bronchiolitis cases represent a complete and pragmatic measure of the real-world population-level impact of RSV immunization. To better inform future policy decisions, addressing this question requires a multinational framework that enables the comparison of the diverse approaches.

In this context, the present study aimed to assess the association between nirsevimab coverage among target populations and the variability in bronchiolitis reduction observed in PEDs across Europe.

## Methods

### Study design and population

We conducted a quasi-experimental controlled interrupted time series analysis, based on the data from the EPISODES multinational study network [17] (see S3 Text).

Twenty-seven PEDs (see Appendix A in S1 Text) in 12 European countries (Austria, France, Hungary, Ireland, Italy, Latvia, Portugal, Spain, Sweden, the Netherlands, Turkey and the United Kingdom) participated in this study.

All bronchiolitis cases aged under 1 year old presenting to one of the participating PEDs between January 1st 2018 and March 31st 2024 were included in the study.

We used urinary tract infections cases aged under 1 year old as a control outcome.

### Data collection

We retrospectively collected monthly aggregated and anonymized data for each participating center through a clinical report form including: age category (0–3 months old = until 12-weeks old; and 3- to 12-months old); diagnosis (bronchiolitis or urinary tract infections; which served as a control outcome); and clinical management (discharged home or admission). As in previous works from this research group [17,18], the identification of patients was based on the ICD-10 coding system (see Appendix B in S1 Text) or on the diagnosis provided by the PED physician in the health record of the case. Consistency in coding practices across and within study sites was monitored at the beginning of the study and overtime. The number of tests performed and RSV positivity rates were obtained through the laboratory databases of a subgroup of centers. The availability of data for each center is provided in Appendix C in S1 Text. All data were stored into secure and validated REDCap electronic data capture tools [19].

STROBE (Observational studies) and RECORD (Routinely-collected health data) reporting guidelines were followed in this study (See S1 Checklist).

### Definition of the intervention groups and periods

In this controlled interrupted time series analysis, centers were classified based on whether or not their region had implemented nirsevimab during the *intervention* period. Thus, we first defined two time periods: the *baseline* period (January 2018 to September 2023) and the *intervention* period (October 2023 to March 2024).

We also defined two study groups:

- The *nirsevimab* group: centers from regions implementing nirsevimab during the *intervention* period, i.e., Paris region (4 centers), Basque Country (1 center), Madrid region (1 center) and Catalonia (1 center).
- The *control* group: centers within regions not implementing nirsevimab during the *intervention* period (20 centers in 10 countries)

### Nirsevimab implementation during the 2023–2024 winter

The guidelines regarding nirsevimab implementation in the Paris Region, Basque Country, Madrid Region and Catalonia are provided in the Table 1. Based on those guidelines, two target populations were identified: infants born “in season”, meaning during the RSV season, for which the immunization was recommended at birth and infants born “out of season”, for which various catch-up immunization programs were implemented. We defined coverage as the number of infants receiving nirsevimab divided by the number of eligible infants. The coverage in each target population (born in-season or out-of-season) across regions was obtained from official reports by health agencies [20,21] or from published data [22–24].

**Table 1:** Immunization strategies implemented during the 2023–2024 season.

	Dates of the Immunization campaign	Population	Overall coverage**
**Paris Region** (see ref 24)	15^th^ September 2023 to 28^th^ February 2024	Overall infants	28%
		Born in season	69%
		Born out of season*	14%
**Basque Country** (See ref 21)	15^th^ October 2023 to 31^st^ March 2024	Overall	50%
		Born in season	90%
		Born out of season*	35%
**Madrid Region** (See ref 20)	1^st^ October 2023 to 31^st^ March 2024	Overall	83%
		Born in season	97%
		Born out of season*	77%
**Catalonia** (See ref 22 and 24)	1^st^ October 2023 to 31^st^ March 2024	Overall	89%
		Born in season	92%
		Born out of season*	87%

* The “born out of season” population was defined as infants aged under 6 months at the beginning of the *intervention* period in Spain; and as infants born between February 7th 2023 and September 14th in France.

** The coverage was defined as the number of infants immunized divided by the number of infants in the targeted population during the intervention period.

To note, during the 2023–2024 RSV season, none of the participating regions had implemented the anti-RSV maternal vaccine [5].

### Outcome measure

The main outcome of the study was the monthly number of bronchiolitis cases presenting to the PEDs over the study period in the 0–3 months and 3–12 months age-groups, assessed by controlled interrupted time-series analysis in the 4 regions from the *nirsevimab* group and the *control* group. These two age-groups were defined based on the hypothesis that nirsevimab implementation for infants born “in season” should drive 0–3 months bronchiolitis epidemiology, while nirsevimab implementation for infants born “out of season” should drive the 3–12 months bronchiolitis epidemiology (see Appendix D in S1 Text). To note, two centers from the *control* group (POR002 and POR004) were excluded from the main analysis because of the unavailability of age categories data.

Secondary outcomes included (i) the monthly number of hospitalized bronchiolitis cases aged under one year, (ii) the monthly number of bronchiolitis cases aged under one year discharged from the PED and (iii) the monthly RSV positivity rate in the centers from the *nirsevimab* group and the *control* group.

### Control outcome

The monthly number of urinary tract infection cases presenting to the PEDs and identified using ICD-10 codes (see Appendix B in S1 Text) was used as a control outcome as it is not expected to be influenced by the implementation of nirsevimab or other seasonal drivers as bronchiolitis.

### Statistical analysis

The main analysis aimed at assessing the correlation between the coverage of nirsevimab and the overall reduction in the monthly number of bronchiolitis cases during the *intervention* period in the 4 regions from the *nirsevimab* group.

To achieve this, we first assessed the evolution of monthly bronchiolitis cases for each of the 4 regions of the *nirsevimab* group using interrupted time series analysis models controlled on the evolution of bronchiolitis cases in the *control* group. Quasi-Poisson regression was used to account for overdispersion in the count data [25–27]. Seasonality was modeled with Fourier terms (sine and cosine functions with 12- and 6-month periods) [25–27]. The secular trend was modeled using a continuous time variable, and the number of bronchiolitis cases observed in the *control* group was included in the model as an external control [28]. One dummy variable (step-change) accounted for the *intervention* period (from October 2023 to March 2024) [25,26] and one continuous variable accounted for the time since intervention (slope-change). Moreover, another dummy variable accounted for the period of implementation of non-pharmaceutical interventions against SARS-CoV-2 (from April 2020 to March 2021). Model validity was assessed through visual inspection of residuals, autocorrelation functions, and Q-Q plots [27] (see Appendix E in S1 Text). For each region of the *nirsevimab* group, and for the *control* group, we then calculated the overall change during the *intervention* period by estimating the fitted value of the number of observed cases compared to the expected value of the counterfactual scenario—if no intervention had occurred—based on the model parameters for each time point. The percentage of reductions and their 95% confidence intervals were obtained through parametric simulation (10,000 random draws from the normal distribution of model-fitted values for the counterfactual scenario).

The second step was to estimate the correlation between the coverage of nirsevimab and the change in the monthly number of bronchiolitis cases across regions and across age-groups during the *intervention* period using a Pearson correlation test.

The following sensitivity analyses were performed: (i) to account for the heterogenicity in the number of patients included by each center, we performed a Pearson correlation analysis weighted on the number of cases included; (ii) to assess the influence of a potential non-independence between estimates, we conducted a sensitivity analysis excluding the control-country estimates from the Pearson correlation; (iii) in addition, we performed a meta-regression to account for between-center variability; (iv) to relax the assumption of linearity required by Pearson’s correlation, we performed a Spearman’s rank correlation; (v) to account for our number of correlation estimates being small, we performed a Kendall correlation test, which is more appropriate for small samples and non-parametric data; (vi) to assess whether the observed trends were dependent on the choice of the controls, we re-estimated the estimates using non-controlled interrupted time-series models and (vii) to account for overdispersion with a different variance structure than the quasi-Poisson model, we re-estimated the estimates using a negative binomial regression [27]. Two sensitivity analyses were added and performed during the reviewing process: (viii) to account for clustering of observations within control centers, we performed a mixed-effects negative binomial model with center-specific random intercepts; and (ix) heterogeneity in coding practices of bronchiolitis were accounted for by re-assessing the Pearson correlation using lower respiratory tract infections as the outcome.

Secondary analyses were performed between the overall coverage in infants aged under one year and the change in the number of hospitalized bronchiolitis, bronchiolitis discharged home from PEDs and in RSV positivity rates. All secondary outcomes were analyzed using the previously described methodology, however, for to assess the change in RSV positivity rates, we used quasi-binomial regression models with a logit link in the interrupted time series analysis to account for overdispersion in proportions and to constrain predicted values between 0 and 100%.

All statistical tests were two-sided, with *P* < 0.05 considered statistically significant. All analyses were performed using RStudio statistical software, version 2022.07.2 (http://www.R-project.org).

### Patient and public involvement and ethics

Patients and the public were not involved in the design, conduct, reporting, or dissemination plans of this research, as this study was based on routinely collected, anonymized data.

This retrospective observational study involving human participants followed the ethical standards of the Declaration of Helsinki. As this study used routinely collected, anonymized data and involved no direct interaction with participants, informed consent was not required and was therefore neither written nor oral. Following initial approval by the UK Health Research Authority (ID: 284008), all participating sites obtained ethical approval and data sharing agreements from their national/local institutional review boards for aggregate data.

### Use of artificial intelligence

ChatGPT (OpenAI; GPT-5.5) was used to assist with R script debugging and language editing. All outputs were reviewed and verified by the authors, who retained full responsibility for the study design, analyses, interpretation, and final manuscript.

## Results

### General characteristics of the population

A total of 107,088 infants younger than 1-year old and diagnosed with a bronchiolitis were included during the overall study period (90,619 between January 2018 and September 2023; and 16,469 between October 2023 and March 2024). Among them, 45,904 (42.9%) and 61,184 (57.1%) were included by participating PEDs from the *nirsevimab* and *control* group respectively. Bronchiolitis cases aged under 3-months accounted for 36.6% (37,764/103,138) of the cases during the study period. Overall, 33.3% of cases (31,755/95,381) required a hospital admission (see Table 2).

**Table 2:** Characteristics of the population, *N* = 124,895.

	PARIS REGION		BASQUE COUNTRY		MADRID REGION		CATALONIA		CONTROL COUNTRIES	
	Baseline period	Intervention period	Baseline period	Intervention period	Baseline period	Intervention period	Baseline period	Intervention period	Baseline period	Intervention period
**Bronchiolitis cases** **0–1 year**	25,634	3,937	3,803	355	3,608	456	7,318	793	50,256	10,928
0–3 months* **(*N*, % of cases)**	7,734/25,634 (30.2%)	1,028/3,937 (26.1%)	1,137/3,803 (29.9%)	79/355 (22.2%)	1,322/3,608 (36.6%)	127/456 (27.8%)	2,669/7,318 (36,4%)	244/793 (30.7%)	19,433/47,365 (41.0%)	3,991/9,867 (40,4%)
3–12 months* **(*N*, % of cases)**	17,900/25,634 (69.8%)	2,909/3,937 (73.9%)	2,666/3,803 (70.1%)	276/355 (77.8%)	2,286/3,608 (63.4%)	329/456 (72.2%)	4,649/7,318 (63.6%)	549/793 (69.3%)	27,932/47,365 (59.0%)	5,876/9,867 (59.6%)
Discharged from PEDs cases** **(*N*, % of cases)**	17,363/25,634 (67.7%)	2,662/3,937 (67.6%)	2,697/3,803 (70.9%)	273/355 (76.9%)	2,292/3,608 (63.5%)	329/456 (72.1%)	4,709/7,318 (64.3%)	516/793 (65.1%)	26,605/40,910 (65.0%)	5,631/8,565 (65.7%)
Admitted cases** **(*N*, % of cases)**	8,134/25,634 (31.7%)	1,273/3,937 (32.3%)	1,071/3,803 (28.2%)	82/355 (23.1%)	1,314/3,608 (36.4%)	127/456 (27.8%)	2,592/7,318 (35.4%)	276/793 (34.8%)	13,956/40,910 (34.1%)	2,930/8,565 (34.2%)
RSV positive tests***	8,134/25,634 (31.7%)	171/763 (22.4%)	766/2,100 (36.5%)	60/529 (11.3%)	1,198/2,186 (54.8%)	112/273 (41.0%)	3,730/17,887 (20.8%)	138/1,942 (7.1%)	7,119/50,436 (14,1%)	2,170/10,356 (20.9%)
**Urinary tract infections cases 0–1 year**	3,571	299	1325	101	280	23	1813	160	9,344	891

RSV: Respiratory Syncytial Virus.

* 0–3 months corresponds to infants aged under 12 weeks-old. Data on the age-group category of bronchiolitis cases were unavailable for two centers in Portugal (POR003 and POR004).

** Data on the outcome of bronchiolitis cases were unavailable for one center in Italy (IT001) and two centers in Portugal (POR003 and POR004).

*** Data on the number of RSV test performed and positivity were only available for: one center in France (FR003), all centers from Spain (SP001,2,3), one center from Ireland (IRE003), two centers from Italy (IT002,6), one center from the Netherlands (NL002), one center from Sweden (SWE001), one center from Turkey (TUR001) and one center from the United Kingdom (UK002).

The baseline period is defined as January 1st 2018 to September 31st 2023.

The nirsevimab period is defined as October 1st 2023 to March 31st 2024

### Evolution of the number of bronchiolitis cases in the *nirsevimab* group

**Fig 1:**
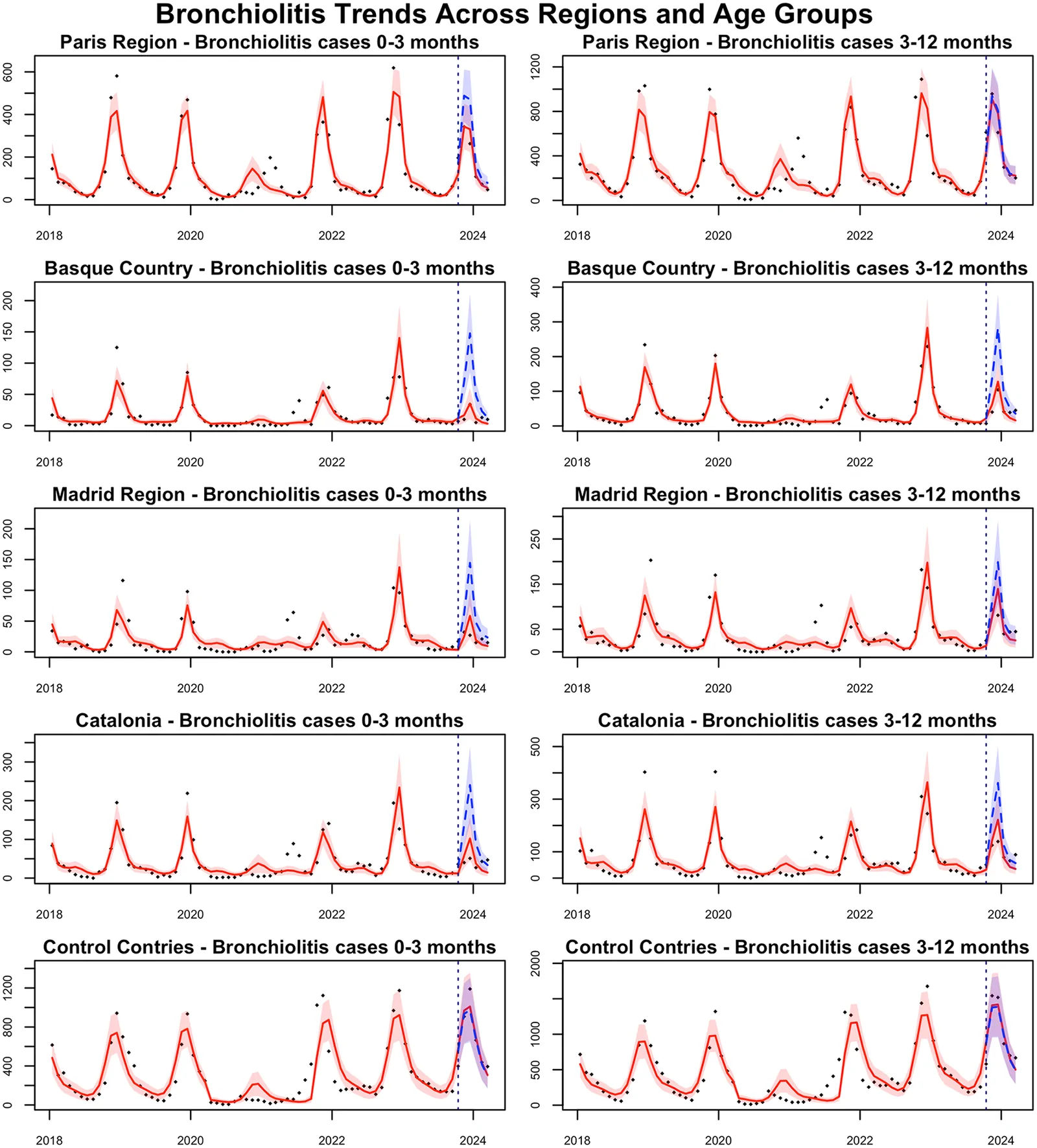
Evolution of the monthly number of bronchiolitis cases across regions (Control countries, Paris Region, Basque Country, Madrid Region and Catalonia) and age-groups (0–3 months and 3–12 months) from January 1st 2018 to March 31st 2024. The black points show the observed data. The vertical dotted blue line corresponds to beginning of the *intervention* period. The solid red line shows the model estimates based on observed data (quasi-Poisson regression modeling) and is associated to its 95%CI in transparent red. The dashed blue line shows the expected values if nirsevimab had not been implemented during the *intervention* period (quasi-Poisson regression modeling) and is associated to its 95%CI in transparent blue.

During the *intervention* period, the monthly number of bronchiolitis cases presenting to PEDs varied across regions of implementation and across targeted population ranging from +0.6% (95% CI [−9.4, +20.5]) in infants aged 3–12 months in the Paris Region to −61.0% (95% CI [−67.8, −35.2]) in infants aged 0–3 months in the Basque country (see Table 3 and Fig 1). Consistently, the reduction in the number of bronchiolitis cases was higher in children aged 0–3 months compared to 3–12 months (see Table 3 and Fig 1).

**Table 3:** Cumulative changes in the number of cases presenting to pediatric emergency departments during the intervention period, assessed by controlled interrupted time series analysis.

		PARIS REGION N = 29,571	BASQUE COUNTRY N = 4,158	MADRID REGION N = 4,086	CATALONIA N = 8,111	CONTROL COUNTRIES N = 61,184
Bronchiolitis cases	0–3 months* N = 37,764	−25.5% [−33.0; −10.4]	−61.0% [−67.8; −35.2]	−54.0% [−61.1; −22.6]	−53.8% [−59.8; −36.0]	+3.6% [−7.9; +29.9]
	3–12 months* N = 65,372	+ 0.6% [−9.4; +20.5]	−40.3% [−47.7; −21.1]	−21.4% [−32.8; +22.3]	−33.1% [−41.2; −12.1]	+2.3% [−8.4; +24.4]
	0–1 years* N = 107,088	−6.1% [−15.4; +12.0]	−44.0% [−51.6; −22.5]	−32.4% [−42.0; +2.5]	−39.6% [−47.0; −19.5]	+13.3% [+2.0; +36.0]
	Discharged from PEDs** N = 63,077	−3.0% [−12.7; +16.1]	−45.0% [−52.4; −24.3]	−3.7% [−34.2; +11.8]	−41.3% [−48.3; -23.6]	−2.3% [−12.2; +17.8]
	Hospitalized** N = 31,755	−15.8% [−24.4; +1.9]	−52.0% [−63.1; −29.4]	−52.5% [−61.2; −2.0]	−41.3% [−49.1; −16.0]	+ 16.4% [+2.9; +48.7]
RSV positivity rates*** N = 90,877 tests		−35.2% [−50.0; +37.5]	−45.0% [−51.5; −25.6]	−45.0% [−51.5; −25.6]	−55.4% [−156.7; +88.4]	+1% [−9.0; +23.5]
Urinary tract infection cases N = 17,807		+7.6% [+1.1; +16.9]	+8.1% [−1.2; +24.6]	−2.5% [−12.1; +16.4]	+ 5.5% [+0.1; +13.0]	+ 3.1% [−0.7; +7.9]

RSV: Respiratory Syncytial Virus.

Changes are reported in percentage, associated to their 95% confidence interval.

* 0–3 months corresponds to infants aged under 12 weeks-old. Data on the age-group category of bronchiolitis cases were unavailable for two centers in Portugal (POR003 and POR004).

** Data on the outcome of bronchiolitis cases were unavailable for one center in Italy (IT001) and two centers in Portugal (POR003 and POR004).

*** Data on the number of RSV test performed and positivity were only available for: one center in France (FR003), all centers from Spain (SP001,2,3), one center from Ireland (IRE003), two centers from Italy (IT002,6), one center from the Netherlands (NL002), one center from Sweden (SWE001), one center from Turkey (TUR001) and one center from the United Kingdom (UK002).

### Control countries and control outcome

No significant change was observed during the *intervention* period, regarding the evolution of the monthly number of bronchiolitis cases aged 0–3 and 3–12 months old in the *control* group, respectively, +3.6% (95% CI [−7.9, +29.9]) and +2.3% (95% CI [−8.4, +24.4]) (see Table 3 and Fig 1).

**Fig 2:**
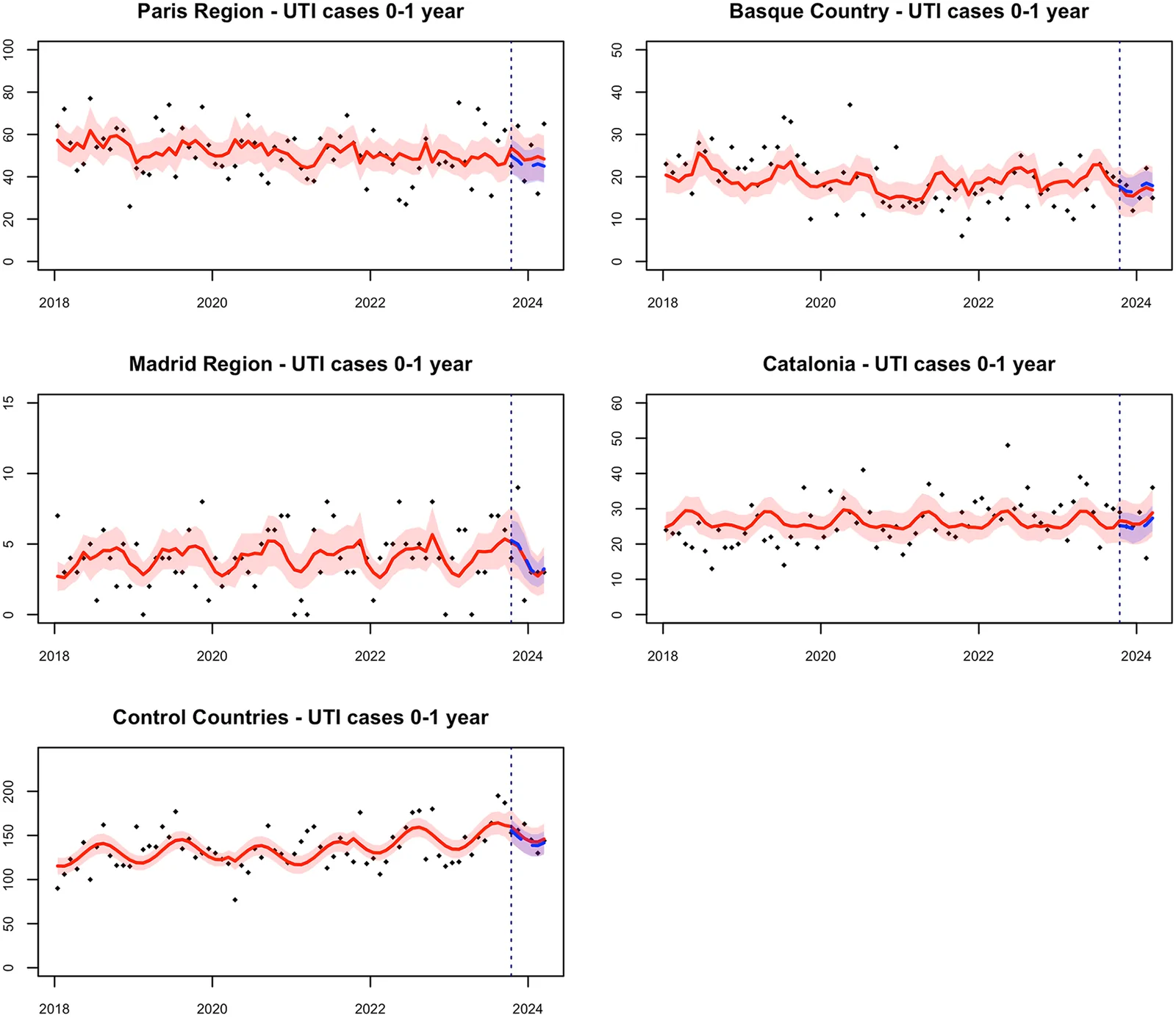
Evolution of the monthly number of cases of the control outcome, urinary tract infections (UTI) in infants (aged 0–1 year), across regions (Control countries, Paris Region, Basque Country, Madrid Region and Catalonia) from January 1st 2018 to March 31st 2024. The black points show the observed data. The vertical dotted blue line corresponds to beginning of the *intervention* period. The solid red line shows the model estimates based on observed data (quasi-Poisson regression modeling) and is associated to its 95%CI in transparent red. The dashed blue line shows the expected values if nirsevimab had not been implemented during the *intervention* period (quasi-Poisson regression modeling) and is associated to its 95%CI in transparent blue.

Besides, no significant change was observed during the *intervention* period in the number of cases of urinary tract infection cases across regions from the *nirsevimab* group and in the *control* group (see Table 3 and Fig 2 and Appendix F in S1 Text).

### Correlation between nirsevimab coverage and the reduction in bronchiolitis cases

**Fig 3:**
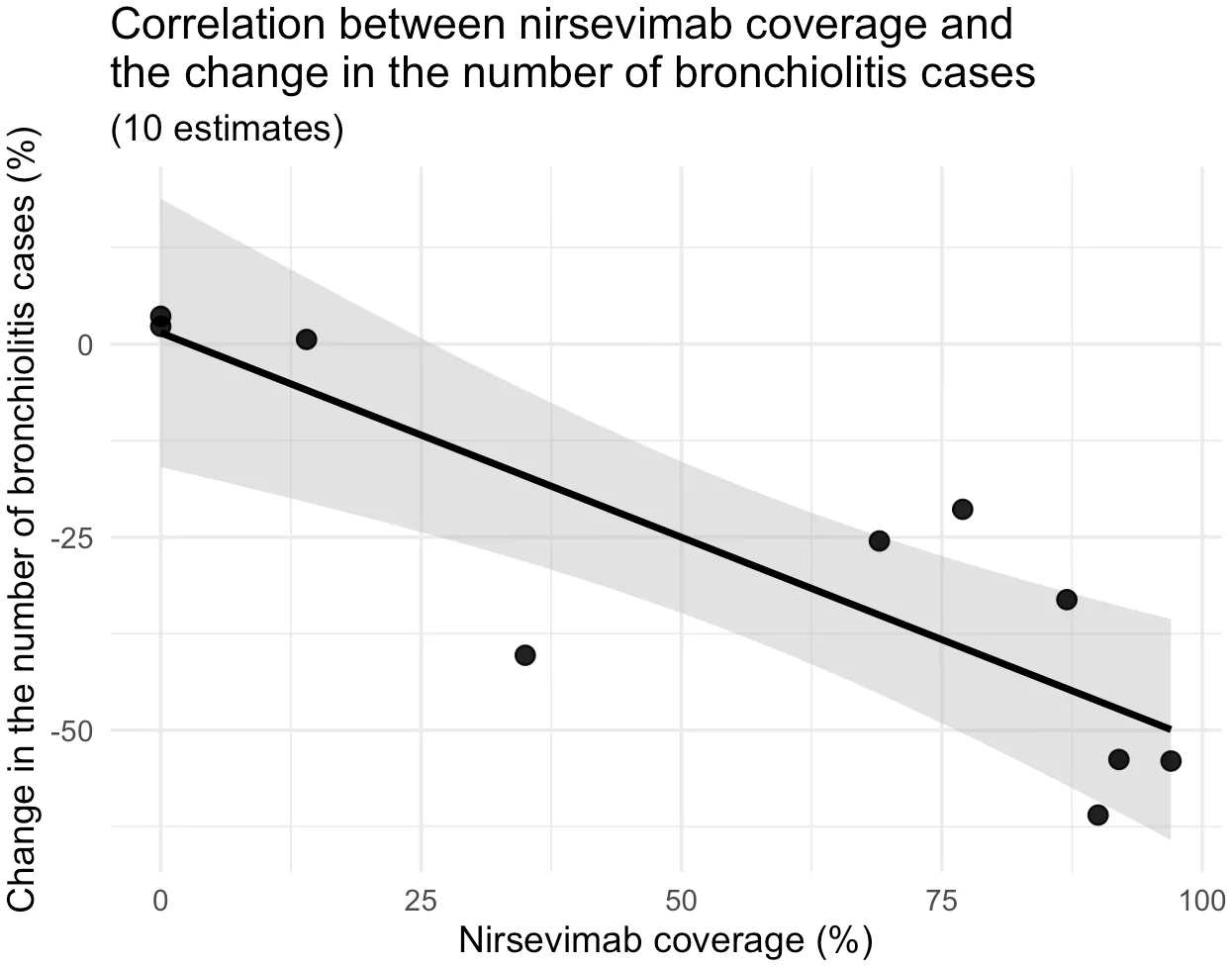
Correlation between the level of coverage of infants born in-season and out-of-season and the percentage of change in bronchiolitis cases across age-groups (0–3 months and 3–12 months) during the nirsevimab period. For each region of the intervention group (*N* = 4) and the control group, one estimate of the cumulative change during the 2023–2024 season was assessed for each age-group (0–3 and 3–12 months). Those 10 estimates were correlated to the level of coverage of the target-group in each region.

We found a strong negative correlation between the level of coverage of infants born in-season and out-of-season and the percentage of change in the number of bronchiolitis cases in the 0–3 months and 3–12 months age-groups, Rho: −0.86 (95% CI [−0.96, −0.50], *p*-value: 0.001). Sensitivity analysis found similar results (see Table 4, Fig 3 and Appendix G in S1 Text).

**Table 4:** Correlation between coverage and the estimated reduction in cases of bronchiolitis during the *intervention* period assessed using a Pearson correlation test and sensitivity analyses.

	Correlation estimates and 95% CI	P-VALUE
MAIN ANALYSIS		
PEARSON CORRELATION TEST BETWEEN THE COVERAGE OF INFANTS BORN IN-SEASON AND OUT-OF-SEASON AND THE PERCENTAGE OF CHANGE OF BRONCHIOLITIS IN THE 0–3 MONTHS AND 3–12 MONTHS AGE-GROUPS DURING THE *INTERVENTION* PERIOD *(N = 10 ESTIMATES)*	−0.86 [−0.96; −0.50]	0.001
SENSITIVITY ANALYSES		
(I) WEIGHTED PEARSON CORRELATION TEST *(N = 10 ESTIMATES)*	−0.93 [−0.99; −0.34]	0.01
(II) PEARSON CORRELATION TEST WITHOUT CONTROL COUNTRIES *(N = 8 ESTIMATES)*	−0.72 [−0.95; −0.04]	0.04
(III) META-REGRESSION TEST *(N = 10 ESTIMATES)*	−0.49 [−0.75; −0.21]	<0.001
(IV) SPEARMAN CORRELATION TEST *(N = 10 ESTIMATES)*	−0.87 [−0.99; −0.47]	<0.001
(V) KENDALL CORRELATION TEST *(N = 10 ESTIMATES)*	−0.72 [−0.98; −0.33]	0.004
(VI) CLASSIC UNCONTROLLED INTERRUPTED TIME SERIES MODEL *(N = 10 ESTIMATES)*	−0.83 [−0.95; −0.43]	0.002
(VII) NEGATIVE BINOMIAL REGRESSION INTERRUPTED TIME SERIES MODEL *(N = 10 ESTIMATES)*	−0.80 [−0.95; −0.35]	0.005
(VIII) MIXED-EFFECTS NEGATIVE BINOMIAL INTERRUPTED TIME SERIES MODEL WITH CENTER-SPECIFIC RANDOM INTERCEPTS FOR THE CONTROL ESTIMATES *(N = 10 ESTIMATES)*	−0.85 [−0.96; −0.47]	0.001
(IX) PEARSON CORRELATION TEST BETWEEN THE COVERAGE OF INFANTS BORN IN-SEASON AND OUT-OF-SEASON AND THE PERCENTAGE OF CHANGE OF LOWER RESPIRATORY TRACT INFECTION CASES IN THE 0–3 MONTHS AND 3–12 MONTHS AGE-GROUPS DURING THE *INTERVENTION* PERIOD *(N = 10 ESTIMATES)*	−0.87 [−0.97; −0.52]	0.001
SECONDARY ANALYSES		
CORRELATION BETWEEN THE OVERALL COVERAGE OF INFANTS AND THE PERCENTAGE OF CHANGE OF HOSPITALIZED BRONCHIOLITIS CASES AGED UNDER 1 YEAR DURING THE *INTERVENTION* PERIOD *(N = 5 ESTIMATES)*	−0.86 [−0.99; +0.06]	0.05
CORRELATION BETWEEN THE OVERALL COVERAGE OF INFANTS AND THE PERCENTAGE OF CHANGE OF DISCHARGED BRONCHIOLITIS CASES AGED UNDER 1 YEAR DURING THE *INTERVENTION* PERIOD *(N = 5 ESTIMATES)*	−0.72 [−0.98; + 0.43]	0.16
CORRELATION BETWEEN THE OVERALL COVERAGE OF INFANTS AND THE PERCENTAGE OF CHANGE IN RSV POSITIVITY RATES DURING THE *INTERVENTION* PERIOD *(N = 5 ESTIMATES)*	−0.78 [−0.98; +0.31]	0.11

RSV, Respiratory Syncytial Virus.

### Secondary outcomes

The results of the secondary analysis are presented in Table 3 and Appendix H to K in S1 Text. As observed for the primary outcome, the estimates of the reduction of the secondary outcomes varied across region of implementation. As an example, we observed a reduction in the RSV positivity rates during the *intervention* period, ranging from −35.2% (95% CI [−50.0, +37.5]) in the Paris region to −55.4% (95% CI [−156.7, +88.4]) in the Basque country, while it remained stable during this period in the control countries, +1% (95% CI [−9.0, +23.5]). The correlation tests for the secondary outcomes also found a negative correlation trend between the estimates and the coverage of nirsevimab across regions (see Table 3 and Appendix K in S1 Text).

## Discussion

This multinational quasi-experimental controlled interrupted time-series analysis found a strong correlation between the level of coverage of nirsevimab in the population of infants born in- and out-of-season and the decrease in bronchiolitis cases aged 0–3 and 3–12 months presenting to PEDs across European countries, respectively.

Before the implementation of nirsevimab, modeling [24] studies warned that discrepancies in targeted population or immunization season could highly modify the impact of nirsevimab. However, during this first year of implementation in Europe, neither the targeted populations nor the timing of nirsevimab immunization varied across regions. By contrast, the coverage was highly variable across age categories and regions implementing nirsevimab, and we found it to be correlated to the level of reduction in the monthly number of bronchiolitis cases presenting to PEDs. This correlation was supported by sensitivity analyses. Moreover, the association was also observed between coverage and the reduction in the number of hospitalized bronchiolitis as well as the RSV positivity rate. Thanks to its multinational design, this study provides valuable insights by showing that coverage emerges as a key factor associated to the heterogeneous reduction in bronchiolitis cases after nirsevimab introduction, despite similar implementation guidelines across Europe during the 2023–2024 season.

This study underscores the importance of understanding the multiple factors shaping uptake (local recommendations, pricing, availability, parental acceptability, etc. [29]) in order to translate clinical efficacy into real-world population benefits. First, both France and Spain showed lower coverage in the population of infants born out-of-season compared to those born in-season. Infants born in-season were offered the immunization directly in the maternity ward during RSV season. This setting, providing a direct access to the parents of a new born, especially during the peak season of bronchiolitis, seems favorable compared to the catch-up immunization program.

Regarding the catch-up strategies, in both France and Spain, nirsevimab immunization was free of charge for the patient during the study period. However, the degree of proactive outreach to families to immunize out-of-season infants was very different between France and the different regions of Spain. The different autonomous regions of Spain would actively invite the parents, by text or emails, to have their child immunized, whereas in France, the proposition relied more heavily on the primary care physician’s recommendations. These organizational differences likely contributed to the heterogeneity in coverage observed across regions. In addition, France experienced a shortage of nirsevimab doses for out-of-season births only two weeks after the start of the immunization campaign. This shortage likely reflected higher-than-anticipated demand for nirsevimab. This poor coverage in infants born out-of-season (14%) was reflected in the stable monthly number of bronchiolitis cases aged 3–12 months presenting to Paris Region PEDs in our study (+0.6%, 95% CI [−9,4; +20.5]). Overall, our findings suggest that the real-world impact of nirsevimab may depend as much on implementation choices as on the product’s intrinsic efficacy.

Moreover, beyond its real-world effectiveness on RSV-bronchiolitis across the full spectrum of disease severity [6,10,14], its broader impact on RSV-related infections such as acute otitis media [30,31] has recently been highlighted. Taking this into account alongside the results from our study, prioritizing specific target populations with nirsevimab could influence the impact of nirsevimab on age-related healthcare resource utilization. Specifically, prioritizing infants born in-season would primarily affect the 0–3 months age group, which accounts for the highest RSV burden in hospital settings [32]. Conversely, prioritizing infants born out-of-season would impact most the RSV-related burden in ambulatory care [6,30] and antibiotic use [33]. Overall, this underscores the critical need of defining the clear public health objectives of nirsevimab immunization to maximize its impact.

This study has several limitations. First, this is an observational study, so a definitive causal relationship between the level of coverage of nirsevimab and the reduction in bronchiolitis cases at PEDs cannot be formally established. Second, the identification of cases was based on the ICD-10 codes of the emergency department record and cases were not reviewed manually. However, these codes have been previously evaluated for the surveillance of bronchiolitis [34]. Moreover, the coding and extraction methodology remained unchanged throughout the study period in the different centers; which is a critical point in interrupted time series analysis. Third, cofounding factors not related to nirsevimab outcome (year-to-year variation, late impact of non-pharmaceutical intervention implementation, etc.) could be associated to changes in the consultation patterns and epidemiology of bronchiolitis cases at PEDs. Using a controlled interrupted time-series methodology allowed to assess the evolution of bronchiolitis cases in the *nirsevimab* group taking into account the evolution of the outcome in the *control* group. Moreover, no change was observed in the evolution of urinary tract infection cases which served as a control outcome. Fourth, factors related to nirsevimab implementation and influencing RSV epidemiological pattern, such as the emergence of resistant strains to nirsevimab could have influenced our results. However, although some new resistant strains of RSV-B have been recently discovered [35], worldwide RSV surveillance systems have not observed any sign of unusual outbreaks recently [36,37]. Fifth, we hypothesized that the coverage for infants born “in season” would drive the 0–3 months bronchiolitis epidemiology, while the one of “out of season” would drive the 3–12 months bronchiolitis epidemiology. Although these age categories can be discussed, they are used in the real-life management of bronchiolitis every day at PEDs across Europe. Sixth, as no monthly coverage of nirsevimab were available in the regions participating in the study, we accounted for it as a static number (coverage achieved by the end of the immunization season) in the correlation analysis and not in the controlled interrupted time series analysis model. Besides, coverage data may not precisely represent the coverage achieved among the specific population served by each participating PED, as PED-level coverage data were unavailable. This is an inherent limitation of real-world studies conducted at such scale. Seventh, RSV is not the only virus responsible for bronchiolitis. To ensure that we were measuring the association between RSV-bronchiolitis and the anti-RSV strategy, we also assessed the evolution of the RSV positivity rates in the regions from the *nirsevimab* group and the control group. Eighth, we did not have access to individual-level data on nirsevimab administration and relied on coverage across targeted populations. This was consistent with the objective of our study, which was to evaluate the association between coverage and bronchiolitis reduction at a population-level rather than the individual-level effectiveness or duration of protection. While individual-level analyses are required to characterize protection over time or potential waning of effectiveness, our ecological approach provides a real-world assessment of the association between program implementation and public health benefit.

This multinational interrupted time-series analysis shows heterogeneous impact of nirsevimab on bronchiolitis across European PEDs, identifying coverage as a key factor associated to the reduction. Our results suggest that implementation choices likely influence the population-level benefit through their effect on coverage. These findings highlight that the benefits of nirsevimab depend not only on its intrinsic efficacy, but also on how efficiently it is delivered in real-world settings.

## Supporting information

S1 Text Supplementary Appendices A–K. (DOCX)

S2 Text RStudio script of the statistical analysis. (R)

S3 Text Resync 3 study protocol. (DOCX)

S1 Checklist RECORD guidelines. Benchimol EI, Smeeth L, Guttmann A, Harron K, Moher D, Petersen I, Sørensen HT, von Elm E, Langan SM, the RECORD Working Committee. The REporting of studies Conducted using Observational Routinely-collected health Data (RECORD) Statement. PLoS Medicine 2015;in press. *Checklist is protected under Creative Commons Attribution (CC BY) license.
(PDF)

S1 Acknowledgments Membership of the EPISODES study Group. (DOCX)

## Abbreviations

PEDs: pediatric emergency department
RSV: Respiratory Syncytial Virus
