# Supplementary material for: Association between nirsevimab coverage and pediatric emergency department visits for bronchiolitis in 12 European countries: An interrupted time-series analysis
Source: PLoS Med. 2026 Sep 3;23(9):e1005223. doi: 10.1371/journal.pmed.1005223 (PMC13568497; doi:10.1371/journal.pmed.1005223)
Supplement: Supplementary file 2 [file pmed.1005223.s001.docx]

APPENDIX

[Appendix n°1: List and characteristics of the 12 participating countries and 27 participating centres	2](#_Toc232413716)

[Appendix n°2: List of ICD-10 codes used to identify acute bronchiolitis and urinary tract infections cases.	3](#_Toc232413717)

[Appendix n°3: Data availability by centre.	4](#_Toc232413718)

[Appendix n°4: Study timeline and age-group definitions in relation to the nirsevimab immunization campaign	5](#_Toc232413719)

[Appendix n°5: Correlograms of the interrupted time series models for 0-3- and 3-12-months age-groups across regions.	6](#_Toc232413720)

[Appendix n°6: Evolution of bronchiolitis and urinary tract infection cases aged under one from January 1^st^ 2018 to March 31^st^ 2024 in the four intervention regions.	11](#_Toc232413721)

[Appendix n°7: Cumulative changes in the number of lower respiratory tract cases presenting to paediatric emergency departments during the intervention period, assessed by controlled interrupted time series analysis	12](#_Toc232413722)

[Appendix n°8: Evolution of the monthly number of admitted bronchiolitis cases in infants aged under 1 year across regions from January 1^st^ 2018 to March 31^st^ 2024.	13](#_Toc232413723)

[Appendix n°9: Evolution of the monthly number of discharged (a) and hospitalized (b) bronchiolitis cases in infants aged under 1 year across regions from January 1^st^ 2018 to March 31^st^ 2024.	14](#_Toc232413724)

[Appendix n°10: Evolution of the monthly percentage of RSV positive respiratory tests performed across regions from January 1st 2018 to March 31st 2024.	16](#_Toc232413725)

[Appendix n°11: Correlation between the level of coverage of infants and the percentage of change in hospitalized bronchiolitis cases aged under one-year(a), discharged bronchiolitis cases aged under one year (b) and the RSV positivity rate across regions (c).	17](#_Toc232413726)

# Appendix A: List and characteristics of the 12 participating countries and 27 participating centres

| **Country and centre** | **Intervention Group** | **Number of bronchiolitis cases included** |
| --- | --- | --- |
| **AUSTRIA**  Medical University of Vienna, Vienna | Control | 339 |
| **FRANCE**  Robert Debré University Hospital, Paris (FR001)  Louis Mourier University Hospital, Colombes (FR002)  Trousseau University Hospital, Paris (FR003)  Jean Verdier University Hospital, Bondy (FR004) | Nirsevimab | 29571 |
| **HUNGARY**  Heim Pal National Paediatric Institute, Budapest (HUN002) | Control | 287 |
| **IRELAND**  Children's Health Ireland at Crumlin, Dublin (IRE001)  Children's Health Ireland at Temple Street, Dublin (IRE002)  Children's Health Ireland at Tallaght, Dublin (IRE003) | Control  Control  Control | 6727  7402  4179 |
| **ITALY**  University Hospital of Padova, Padova (IT001)  Fondazione Policlinico Universitario A. Gemelli IRCCS, Rome (IT002)  Bambino Gesù Children’s Hospital, IRCCS, Rome (IT005)  Fondazione IRCCS Ca' Granda Ospedale Maggiore Policlinico, Milano (IT006) | Control  Control  Control  Control | 1416  1052  5358  1980 |
| **LATVIA**  Children's Clinical University Hospital, Riga (LAT001) | Control | 1433 |
| **PORTUGAL**  Centro Hospitalar e Universitário de São João, Porto (POR002)  Centro Hospitalar Lisboa Central, Lisbon (POR003)  Hospital Prof. Doutor Fernando da Fonseca, Amadora (POR004) | Control  Control  Control | 2733  6953  5237 |
| **SPAIN**  Cruces University Hospital, Barakaldo (SP001)  Hospital Universitario La Paz, Madrid (SP003)  Hospital Sant Joan de Déu Barcelona (SP004) | Nirsevimab  Nirsevimab  Nirsevimab | 4158  4064  8111 |
| **SWEDEN**  Astrid Lindgrens Children's hospital, Stockholm (SWE001) | Control | 4269 |
| **NETHERLANDS**  ErasmusMC – Sophia, Rotterdam (NL001)  NoordWest Ziekenhuisgroep, Alkmaar (NL002) | Control  Control | 114  529 |
| **TURKEY**  Ondokuz Mayıs University, Samsun (TUR001) | Control | 582 |
| **UNITED** **KINGDOM**  Leicester University, Leicester (UK001)  St. Mary’s hospital, London (UK002)  Bristol Royal Hospital for Children, Bristol (UK005) | Control  Control  Control | 9535  2956  6341 |

# Appendix B: List of ICD-10 codes used to identify acute bronchiolitis and urinary tract infections cases.

- **Acute bronchiolitis**

J21.0: Acute bronchiolitis due to respiratory syncytial virus

J21.1: Acute bronchiolitis due to human metapneumovirus

J21.8: Acute bronchiolitis due to other specified organisms

J21.9: Acute bronchiolitis, unspecified

- **Pneumonia**

J13: Pneumonia due to Streptococcus pneumoniae

J14: Pneumonia due to Hemophilus influenzae

J15.0: Pneumonia due to Klebsiella pneumoniae

J15.1: Pneumonia due to Pseudomonas

J15.2: Pneumonia due to staphylococcus

J15.3: Pneumonia due to streptococcus, group B

J15.4: Pneumonia due to other streptococci

J15.5: Pneumonia due to Escherichia coli

J15.6: Pneumonia due to other Gram-negative bacteria

J15.7: Pneumonia due to Mycoplasma pneumoniae

J15.8: Pneumonia due to other specified bacteria

J15.9: Unspecified bacterial pneumonia

J16: Pneumonia due to other infectious organisms, not elsewhere classified

J16.0: Chlamydial pneumonia

J16.8: Pneumonia due to other specified infectious organisms

J17: Pneumonia in diseases classified elsewhere

J18.0: Bronchopneumonia, unspecified organism

J18.1: Lobar pneumonia, unspecified organism

J18.2: Hypostatic pneumonia, unspecified organism

J18.8: Other pneumonia, unspecified organism

J18.9: Pneumonia, unspecified organism

J85.1: Abscess of lung with pneumonia

J86.0: Pyothorax

J86.9: Pyothorax without fistula

J90: Pleural effusion, not elsewhere classified

- **Urinary tract infections**

N39.0: Urinary tract infection, site not specified

N10: Acute pyelonephritis

N13.6: Pyonephrosis

# Appendix C: Data availability by center.

| **Country and centre** | **DATA AVAILABILITY** | | | |
| --- | --- | --- | --- | --- |
|  | **Bronchiolitis** | | **RSV testing** | **Urinary tract infections** |
|  | **Age-categories** | **Outcome** |  |  |
| **AUSTRIA**  Medical University of Vienna, Vienna | Yes | Yes | No | Yes |
| **FRANCE**  Robert Debré University Hospital, Paris (FR001)  Louis Mourier University Hospital, Colombes (FR002)  Trousseau University Hospital, Paris (FR003)  Jean Verdier University Hospital, Bondy (FR004) | Yes  Yes  Yes  Yes | Yes  Yes  Yes  Yes | No  No  Yes  No | Yes  Yes  Yes  Yes |
| **HUNGARY**  Heim Pal National Paediatric Institute, Budapest (HUN002) | Yes | Yes | No | Yes |
| **IRELAND**  Children's Health Ireland at Crumlin, Dublin (IRE001)  Children's Health Ireland at Temple Street, Dublin (IRE002)  Children's Health Ireland at Tallaght, Dublin (IRE003) | Yes  Yes  Yes | Yes  Yes  Yes | No  No  Yes | Yes  Yes  Yes |
| **ITALY**  University Hospital of Padova, Padova (IT001)  Fondazione Policlinico Universitario A. Gemelli IRCCS, Rome (IT002)  Bambino Gesù Children’s Hospital, IRCCS, Rome (IT005)  Fondazione IRCCS Ca' Granda Ospedale Maggiore Policlinico, Milano (IT006) | Yes  Yes  Yes  Yes | No  Yes  Yes  Yes | No  Yes  No  Yes | Yes  Yes  Yes  Yes |
| **LATVIA**  Children's Clinical University Hospital, Riga (LAT001) | Yes | Yes | No | Yes |
| **PORTUGAL**  Centro Hospitalar e Universitário de São João, Porto (POR002)  Centro Hospitalar Lisboa Central, Lisbon (POR003)  Hospital Prof. Doutor Fernando da Fonseca, Amadora (POR004) | Yes  No  No | Yes  No  No | No  No  No | Yes  Yes  Yes |
| **SPAIN**  Cruces University Hospital, Barakaldo (SP001)  Hospital Universitario La Paz, Madrid (SP003)  Hospital Joan de Deu, Barcelona (SP004) | Yes  Yes  Yes | Yes  Yes  Yes | Yes  Yes  Yes | Yes  Yes  Yes |
| **SWEDEN**  Astrid Lindgrens Children's hospital, Stockholm (SWE001) | Yes | Yes | Yes | Yes |
| **NETHERLANDS**  ErasmusMC – Sophia, Rotterdam (NL001)  NoordWest Ziekenhuisgroep, Alkmaar (NL002) | Yes  Yes | Yes  Yes | No  Yes | Yes  Yes |
| **TURKEY**  Ondokuz Mayıs University, Samsun (TUR001) | Yes | Yes | Yes | Yes |
| **UNITED** **KINGDOM**  Leicester University, Leicester (UK001)  St. Mary’s hospital, London (UK002)  Bristol Royal Hospital for Children, Bristol (UK005) | Yes  Yes  Yes | Yes  Yes  Yes | No  Yes  No | Yes  Yes  Yes |

# Appendix D: Study timeline and age-group definitions in relation to the nirsevimab immunization campaign

# Appendix E: Correlograms of the interrupted time series models for 0-3- and 3-12-months age-groups across regions.

# Appendix F: Evolution of bronchiolitis and urinary tract infection cases aged under one from January 1^st^ 2018 to March 31^st^ 2024 in the four intervention regions.

# Appendix G: Cumulative changes in the number of lower respiratory tract cases presenting to paediatric emergency departments during the intervention period, assessed by controlled interrupted time series analysis

(N=127,041 cases)

|  | PARIS REGION | BASQUE COUNTRY | MADRID REGION | CATALONIA | CONTROL COUNTRIES |
| --- | --- | --- | --- | --- | --- |
| 0-3 months*  N=42,137 | -24.9 %  [-32.4; -9.9]  p-value=0.14 | - 62.1%  [-68.6; -37.7]  p-value<0.001 | - 55.9 %  [-62.8; -26.0]  p-value=0.03 | - 54.6 %  [-60.5; -37.1]  p-value=0.01 | + 8.8 %  [-2.9; +33.9]  p-value=0.69 |
| 3-12 months*  N=89,904 | -2.2 %  [-12.0; +17.1]  p-value=0.90 | - 46.6 %  [-52.8; -32.5]  p-value<0.001 | - 23.7 %  [-34.4; +12.7]  p-value=0.36 | - 39.5 %  [-46.4; -23.6]  p-value=0.03 | + 17.2 %  [6.1; 38.5]  p-value=0.38 |

# Appendix H: Evolution of the monthly number of admitted bronchiolitis cases in infants aged under 1 year across regions from January 1^st^ 2018 to March 31^st^ 2024.

The black points show the observed data. The vertical dotted blue line corresponds to beginning of the *intervention* period. The solid red line shows the model estimates based on observed data (quasi-Poisson regression modelling) and is associated to its 95%CI in transparent red. The dashed blue line shows the expected values if nirsevimab had not been implemented during the *intervention* period (quasi-Poisson regression modelling) and is associated to its 95%CI in transparent blue.

# Appendix I: Evolution of the monthly number of discharged (a) and hospitalized (b) bronchiolitis cases in infants aged under 1 year across regions from January 1^st^ 2018 to March 31^st^ 2024.

(a)

The black points show the observed data. The vertical dotted blue line corresponds to beginning of the *intervention* period. The solid red line shows the model estimates based on observed data (quasi-Poisson regression modelling) and is associated to its 95%CI in transparent red. The dashed blue line shows the expected values if nirsevimab had not been implemented during the *intervention* period (quasi-Poisson regression modelling) and is associated to its 95%CI in transparent blue.

(b)

The black points show the observed data. The vertical dotted blue line corresponds to beginning of the *intervention* period. The solid red line shows the model estimates based on observed data (quasi-Poisson regression modelling) and is associated to its 95%CI in transparent red. The dashed blue line shows the expected values if nirsevimab had not been implemented during the *intervention* period (quasi-Poisson regression modelling) and is associated to its 95%CI in transparent blue.

Appendix J: Evolution of the monthly percentage of tests positive for Respiratory Syncytial Virus (RSV) across regions from January 1st 2018 to March 31st 2024.

The black points show the observed data. The vertical dotted blue line corresponds to beginning of the *intervention* period. The solid red line shows the model estimates based on observed data (quasi-Poisson regression modelling) and is associated to its 95%CI in transparent red. The dashed blue line shows the expected values if nirsevimab had not been implemented during the *intervention* period (quasi-Poisson regression modelling) and is associated to its 95%CI in transparent blue.

# Appendix K: Correlation between the level of coverage of infants and the percentage of change in hospitalized bronchiolitis cases aged under one-year(a), discharged bronchiolitis cases aged under one year (b) and the RSV positivity rate across regions (c).

(b)

(a)

(c)

RSV: Respiratory Syncytial Virus
