# Supplementary material for: Association between nirsevimab coverage and pediatric emergency department visits for bronchiolitis in 12 European countries: An interrupted time-series analysis
Source: PLoS Med. 2026 Sep 3;23(9):e1005223. doi: 10.1371/journal.pmed.1005223 (PMC13568497; doi:10.1371/journal.pmed.1005223)
Supplement: Supplementary file 3 [file pmed.1005223.s003.docx]

**ReSync Study 3:**

**Time series analysis of the impact of public health measures on respiratory tracts infections in pediatric emergency departments**

**CHIEF INVESTIGATOR:**

Luigi Titomanlio, [luigi.titomanlio@aphp.fr](mailto:luigi.titomanlio@aphp.fr)
University of Paris
Hôpital Robert Debré, APHP, 48 Bd Sérurier
75019 Paris, France

**CO-INVESTIGATORS:**

EPISODES study steering group:

Silvia Bressan, [silviabress@gmail.com](mailto:silviabress@gmail.com) ^1^,

Danilo Buonsenso, [danilobuonsenso@gmail.com](mailto:danilobuonsenso@gmail.com) ^2,3^,

Ruth Farrugia, [ruth.b.farrugia@gov.mt](mailto:ruth.b.farrugia@gov.mt) ^4^,

Niccolo Parri, [niccoparri@gmail.com](mailto:niccoparri@gmail.com) ^5^,

Rianne Oostenbrink, [r.oostenbrink@erasmusmc.nl](mailto:r.oostenbrink@erasmusmc.nl) ^6^,

Damian Roland, [dr98@leicester.ac.uk](mailto:dr98@leicester.ac.uk) ^7,8^,

Ian Maconochie, [i.maconochie@imperial.ac.uk](mailto:i.maconochie@imperial.ac.uk) ^9^,

Liviana Da Dalt, [liviana.dadalt@unipd.it](mailto:liviana.dadalt@unipd.it) ^1^,

Zsolt Bognar, [bognarzs@heimpalkorhaz.hu](mailto:bognarzs@heimpalkorhaz.hu) ^10^

Prof Henriette A Moll, [h.a.moll@erasmusmc.nl](mailto:h.a.moll@erasmusmc.nl) ^6^

Ruud G. Nijman, [ruud.nijman@nhs.net](mailto:ruud.nijman@nhs.net) ^9^

^1^Division of Paediatric Emergency Medicine, Department of Women’s and Children’s Health – University Hospital of Padova, Italy;

^2^Department of Woman and Child Health and Public Health, Fondazione Policlinico Universitario A. Gemelli IRCCS, Rome, Italy

^3^Università Cattolica del Sacro Cuore, Roma, Italia

^4^ Department of Child and Adolescent Health, Mater Dei Hospital, Msida, Malta

^5^ Emergency Department & Trauma Center, Ospedale Paediatrico Meyer Firenze, Florence, Italy.

^6^ Department general Paediatrics, ErasmusMC – Sophia, Rotterdam, The Netherlands

^7^ SAPPHIRE Group, Health Sciences, Leicester University, Leicester, UK

^8^ Paediatric Emergency Medicine Leicester Academic (PEMLA) Group, Leicester Hospitals, Leicester, UK

^9^ Department of Paediatric Emergency Medicine, Division of Medicine, St. Mary’s hospital - Imperial College NHS Healthcare Trust, London, London, London, UK

^10^ Department of Paediatric Emergency Medicine, Heim Pal National Paediatric Institute, Budapest, Hungary

**Statistician**: Kate Honeyford, [k.honeyford@imperial.ac.uk](mailto:k.honeyford@imperial.ac.uk); Global Digital Health Unit, Imperial College London

**SPONSOR**

Imperial College London is the main research Sponsor for this study.

For further information regarding the sponsorship conditions, please contact the Head of Regulatory Compliance at:

Joint Research Compliance Office

Imperial College London and Imperial College Healthcare NHS Trust

Room 215, Level 2, Medical School Building

Norfolk Place

London, W2 1PG

Tel: 0207 594 9459/ 0207 594 1862

<http://www3.imperial.ac.uk/clinicalresearchgovernanceoffice>

1. **Study summary**

| **TITLE** | RESYNC 3: Time series analysis of the impact of public health measures on respiratory tracts infections in pediatric emergency departments |
| --- | --- |
| **DESIGN** | Retrospective analysis of routinely collected clinical data of children presenting to emergency departments across Europe. |
| **AIMS** | We aim to assess and compare the impact of nirsevimab, a new anti-RSV monoclonal antibody, and Abrysvo, a new maternal anti-RSV vaccine, on respiratory tract infections presenting to pediatric emergency departments in Europe. |
| **OUTCOME MEASURES** | Primary outcome: Number of cases of several acute respiratory tract infections (bronchiolitis, pneumonia, otitis media, croup/laryngitis, tonsilitis/pharyngitis) per month during the study period.  Secondary outcome:  - Severity of the diagnosis as defined by the following outcomes: need for hospital admission, PICU, and death.  - RSV positivity rates per month for bronchiolitis and pneumonia  Control outcome: number of cases of urinary tract infections per month |
|  |  |
| **POPULATION** | All consecutive children less than 16 years of age presenting to the emergency department of one of the participating centers during the study period, diagnosed with acute otitis media, laryngitis/croup, tonsillitis/pharyngitis, bronchiolitis, pneumonia and urinary tract infection. |
| **DURATION** | January 1^st^ 2018 to March 30^th^ 2025 |

2. **Study hypothesis**

**Respiratory Syncytial Virus (RSV) is the most common causative agent of RTI** in infants(1), mostly bronchiolitis in children aged under one. RSV is responsible for lower RTI such as bronchiolitis and pneumonia, as well as upper RTI such as acute otitis media. Furthermore, RSV has been shown to trigger specifically non-invasive and invasive pneumococcal diseases, especially pneumococcal pneumonia(2–4).

As of 2023, there was no available preventive treatment against RSV for the general population. In October 2022, **the European Medicines Agency (EMA) approved the first long-lasting monoclonal antibody targeting RSV– nirsevimab (Beyfortus®) – in the prevention of lower RTI in children under 1 year**. In phase III trials for in term and late pre-term infants, nirsevimab reduced by 74.9% (95% CI 50.6, 87.3) the medically attended RSV lower RTI(5). Thus, nirsevimab is indicated in the general population and requires only one injection at the beginning of the RSV season. Following EMA approval, France(6), Spain(7) and Luxembourg were the first countries to implement nirsevimab in the general paediatric population as of September 2023.

Since 2020, our study group have worked on modelling the impact of non-pharmaceutical interventions (NPI) implemented to stop the COVID-19 pandemic, on respiratory tract infections presenting to PEDs in Europe. A first manuscript assessed the respective impact of various NPI on the reduction in RTI cases during the implementation of NPI and a second manuscript assessed the impact of NPI lifting. Indeed, a drastic decrease in RTI cases was observed, increasing the population naïve to respiratory viruses; which secondary led to important surges of RSV cases, when non-pharmaceutical interventions were lifted. Thus, **at a national level, the last years provided a highly unstable epidemiological baseline at the time of implementing nirsevimab and Abrysvo®.** As a consequence, assessing the impact of the preventive strategies at a national scale will be highly challenging. **Multinational surveillance systems with control countries (implementing neither nirsevimab nor Abrysvo®) is the most suitable quasi-experimental design** to estimate the population-level impact of these unique public health interventions(8).

Using controlled interrupted time-series analyses, the objectives of the study is to model the impact, in European PEDs, of nirsevimab RTI and to compare the estimated impact of the various implementation scheme of nirsevimab across European countries.

**Ethics approval**

Amendment to the EPISODES 2 study, as the ReSync study is a substudy from the EPISODES study.

3. **Study design**

**3.1 Design**

**We will conduct controlled interrupted time series analyses with intervention countries** (countries implementing one or both of the anti-RSV immunization strategy) **and control countries** (countries not implementing any of the interventions).

Nirsevimab has been implemented in September 2023 for the first time in 3 European countries: France, Spain and Luxembourg. Abrysvo has been implemented as of September 2024. Based on this, we have constituted two groups: intervention countries (countries implementing one or both of the anti-RSV immunization strategy) and control countries.

- Aggregated, anonymous data will be entered on a monthly basis for each individual participating center;

- Each month or each week will start at the first Monday (00:00 am) of that time period, through to the last Sunday (11:59 pm) of that time period.

**Primary study design:** Observational, retrospective

**Secondary study design**: Epidemiological study

**Trial setting**: Hospitals

**Condition/studied diagnosis**: Acute otitis media, laryngitis/croup, pharyngitis/tonsillitis, bronchiolitis, pneumonia and urinary tract infection.

**3.2 Outcome**

**Primary outcome measure**

Number of acute otitis media, laryngitis/croup, pharyngitis/tonsillitis, bronchiolitis, pneumonia, and urinary tract infection visits per month in the pediatric emergency department of the participating centers

**Secondary outcome measures**
- Severity of the cases as defined by the outcome: hospitalization to short stay unit, to ward, to PICU, death in the PED or discharged home;

- RSV positivity rates for bronchiolitis and pneumonia

**3.3 Data collection**

- A clinical report form has been constructed to capture the anonymised and aggregated data for each participating centre, in a standardized and harmonised manner
- Prior to participation, each centre will indicate which data collection system is being used and which data are routinely available for extraction from their electronic health records
- Minimum requirement of available data for participation include: denominator data of all children presenting, working diagnosis and completion of site specific survey.
- For this study, manual data extraction by the participating institutions has been discouraged to ensure timely, standardized and efficient collection of the data.
- The digital data entry system will account for the availability of data of participating centres using conditional linking and centres will only be asked to entre data that is routinely available in their institution
- Each participating institution will be asked to complete a survey detailing the implementation of nirsevimab (or not) in their region during the intervention period.
- The clinical report forms will collect, for each studied diagnosis, data on:
  - Gender, age
  - Outcome: admission to short stay unit, to ward, to PICU, death or discharged home
  - Rsv positivity: number of tests performed and number of positive tests.
- All data are routinely collected clinical variables as part of current best clinical care recommendations
- Some data have already been retrieved from EPISODES 1, 2 and the ReSync 1,2 study.

**3.4 Data collection and data management**

- Local data will only be accessed by members of the local clinical team; no one outside the local clinical team will have access to any person identifiable data or any data at a patient individual level.
- The aggregated and anonymised data will be entered using the validated online data entry software Redcap

(website portal: <http://redcap.euclids-ci.eu/redcap/index.php?action=myprojects> )

- The site specific survey will use the same platform and storing of data. The site will only be identifiable by a specific site code; the PI has a list of the institutions and their site code; all sites will be given a unique site code for all data entry.
- Data will be stored in a data storage cloud alongside data from other data entry projects of the Section of paediatric infectious diseases at Imperial College.
- Data custodian: Dr. Tisham De, [tisham.de08@imperial.ac.uk](mailto:tisham.de08@imperial.ac.uk), data manager, room 235, Medical School building, St Mary’s campus, Imperial College

4. **Eligibility**

**Participant inclusion criteria**
- All consecutive children presenting to the emergency department of one of the participating centers during the study period, less than 16 years-old, diagnosed with acute otitis media, laryngitis/croup, pharyngitis/tonsillitis, bronchiolitis, pneumonia, and urinary tract infection.

**Participant type:** Patient

**Age group:** 0 to <16 years-old

**Gender:** Both

**Participant exclusion criteria:** none

5. **Assessment and follow-up**

This study will only collect cross-sectional data and the data will only reflect data from the duration of a patient’s stay in the hospital.

There will not be any follow-up or linking of longitudinal data as part of this study.

6. **Statistics and data analysis**

**6.1 Statistical analysis**

- Descriptive analysis, using absolute numbers and percentages, per centre and for all centres combined
- Changes in time will be assessed per countries, and will be described as the change in absolute numbers and percentages.
- Controlled interrupted time series analysis will be conducted to account for seasonality, autocorrelation and secular trend of the outcomes of interest

**6.2 Countries of recruitment**

European countries with centers participating to the EPISODES and REPEM networks

Trial Centre Name : Imperial College
Address City : London
Country : United Kingdom
Zip : W21NY

7. **Results and Publications**

**7.1 Publication and dissemination plan**

Considering the burden of RSV in the landscape of paediatric diseases, these new preventive interventions could completely reshape the epidemiology of paediatric RTI. Our work will assess the impact of nirsevimab and Abrysvo on RTI cases presenting to PED in Europe and compare the respective impact of various implementation scheme of anti-RSV immunisation across countries. This will provide unvaluable information to guide public health decision making about optimal interventions to reduce the burden of RSV-related diseases.

The steering group has extensive experience in this type of research and the use of the European network of pediatric emergency care physicians. (Mintegi et al. 2008; Van de Voorde et al. 2013; van de Maat et al. 2019) This type of research fits in well with the outlined research priorities of the Research in Paediatric Emergency Medicine (REPEM) Network.(Bressan et al. 2019).

We expect to publish our main manuscript in a leading international peer reviewed journal, and to present this work at international conferences.

**Intention to publish date:** Spring 2026

**7.2 Publication policy**

**Authorship agreement**

- Named authors on any manuscript and other research output originating from the ReSync - European Bronchiolitis Study study data will be derived from the study group
- Any manuscript or other research output derived from the ReSync - European Bronchiolitis Study data will be published on behalf of the EPISODES-group, with institutions named in alphabetical order.
- Additional authors may be considered as long as all the international criteria for authorship contribution have been fulfilled; additional authors should be proposed early to the lead investigators by the site lead.
- Authors are responsible for providing their correct affiliations and titles, and on checking this for any final version of any manuscript or other research output, such as conference abstracts, that will be submitted
- The lead investigators are responsible for disseminating any research output to the other members of the EPISODES-group as early as possible for their valued input, checking of correctness and completeness, and their final approval for submission.
- Authors are responsible for signing their (electronic) conflict of interest forms (ICMJE forms, <http://www.icmje.org/conflicts-of-interest/> ) within 72 hours after having been requested to do so. Failure to do so might lead to removal from the authorship list by the lead author.
- Authors can nominate people to be acknowledged for their contribution to the ReSync - European Bronchiolitis Study; the responsibility of informing these people and gaining their consent to be mentioned in the acknowledgement section lies with the site lead of each institution. This is not equivalent to an authorship, and these people will not be listed in the EPISODES study contributors list.
- Sequence of the main authors will be alphabetical, except for the first – second – second last – last authors that will be decided by the lead authors.

**7.3 Publication citation(s) Contact(s)**

**Contacts**
Luigi Titomanlio, MD, PhD, Professor of Pediatrics
Pediatrics Emergency Department

Hôpital Robert Debré, APHP, 48 Bd Sérurier 75019 Paris
Country : France
Tel : +33140034005
Email : [luigi.titomanlio@aphp.fr](mailto:luigi.titomanlio@aphp.fr)

Ruud.G. Nijman, MD
Specialty registrar in Paediatrics
GRID Paediatric Emergency Medicine
NIHR Academic clinical lecturer

Department of Infectious Diseases

Section of Paediatric Infectious Diseases
Imperial College London
St. Mary's Campus
Medical School
Room 235
Norfolk Place
W2 1PG
Paddington, London
Country : UK
telephone: (0044) 07442360730
e-mail: [ruud.nijman@nhs.net](mailto:ruud.nijman@nhs.net)

Website: [https://www.imperial.ac.uk/people/r.nijman](https://clicktime.symantec.com/3JJyFKAUnkScoA7YcPtw41N6H2?u=https%3A%2F%2Fwww.imperial.ac.uk%2Fpeople%2Fr.nijman)

8. **Sponsor(s) and fundings**

**8.1 Sponsor**
Organization : Imperial College London
Address : Joint Research Compliance Office Medical School building, Room 221 Norfolk Place , City , London
Country : United Kingdom
Zip : W211PG
Tel : xxx
Email : cheuk-fung.wong@imperial.ac.uk
Type : University/education
Website : http://www3.imperial.ac.uk/

**8.2 Funder(s)**

**Funding Type** : None

9. **References**

1.	Troeger C, Blacker B, Khalil IA, Rao PC, Cao J, Zimsen SRM, et al. Estimates of the global, regional, and national morbidity, mortality, and aetiologies of lower respiratory infections in 195 countries, 1990–2016: a systematic analysis for the Global Burden of Disease Study 2016. Lancet Infect Dis. nov 2018;18(11):1191‑210.

2.	Ouldali N, Deceuninck G, Lefebvre B, Gilca R, Quach C, Brousseau N, et al. Increase of invasive pneumococcal disease in children temporally associated with RSV outbreak in Quebec: a time-series analysis. Lancet Reg Health - Am. mars 2023;19:100448.

3.	Rybak A, Levy C, Angoulvant F, Auvrignon A, Gembara P, Danis K, et al. Association of Nonpharmaceutical Interventions During the COVID-19 Pandemic With Invasive Pneumococcal Disease, Pneumococcal Carriage, and Respiratory Viral Infections Among Children in France. JAMA Netw Open. 28 juin 2022;5(6):e2218959.

4.	Rybak A, Assad Z, Levy C, Bonarcorsi S, Béchet S, Werner A, et al. Age-specific resurgence in invasive pneumococcal disease incidence in the COVID-19 pandemic era and its association with respiratory virus and pneumococcal carriage dynamics: a time series analysis. Clin Infect Dis Off Publ Infect Dis Soc Am. 6 déc 2023;ciad746.

5.	Muller WJ, Madhi SA, Seoane Nuñez B, Baca Cots M, Bosheva M, Dagan R, et al. Nirsevimab for Prevention of RSV in Term and Late-Preterm Infants. N Engl J Med. 20 avr 2023;388(16):1533‑4.

6.	Haute Autorité de Santé. AVIS SUR LES MEDICAMENTS: nirsévimab, Adopté par la Commission de la transparence le 19 juillet 2023 [Internet]. [cité 19 sept 2023]. Disponible sur: https://www.has-sante.fr/upload/docs/evamed/CT-20356_BEYFORTUS_PIC_INS_AvisDef_CT20356.pdf

7.	Francisco L, Cruz-Cañete M, Pérez C, Couceiro JA, Otheo E, Launes C, et al. Nirsevimab for the prevention of respiratory syncytial virus disease in children. Statement of the Spanish Society of Paediatric Infectious Disease (SEIP). An Pediatr. oct 2023;99(4):257‑63.

8.	Hawkins NG, Sanson-Fisher RW, Shakeshaft A, D’Este C, Green LW. The Multiple Baseline Design for Evaluating Population-Based Research. Am J Prev Med. août 2007;33(2):162‑8.
