# Supplementary material for: Association between nirsevimab coverage and pediatric emergency department visits for bronchiolitis in 12 European countries: An interrupted time-series analysis
Source: PLoS Med. 2026 Sep 3;23(9):e1005223. doi: 10.1371/journal.pmed.1005223 (PMC13568497; doi:10.1371/journal.pmed.1005223)
Supplement: Supplementary file 4 [file pmed.1005223.s005.docx]

**The EPISODES study group**

Léa Lenglart MD^1,2^, Luigi Titomanlio MD PhD^1^, Ilaria Alberti MD^3,4^, Laura Almeida MD^5^, Camille Aupiais MD PhD^6,7^, Esra Akyüz Özkan MD PhD^8^, Michael Barrett MD^9,10^, Romain Basmaci MD PhD^2,11^, Ahmet Birbilen MD^12^, Dorine Borensztajn MD PhD^13^, Catarina Braz Schönenberger MD^14^, Silvia Bressan MD PhD^15^, Danilo Buonsenso MD PhD^16,17^, Teresa Campos MD^5^, Susana Castanhinha MD^18^, Antonio Chiaretti MD PhD^16,17^, Gemma Claret MD PhD^19^, Francesca De Zan MD^15^, Sheena Durnin MD^20,21^, Laszlo Fodor MD^22^, Borja Gomez MD^23^, Susanne Greber-Platzer MD PhD^24^, Romain Guedj MD PhD^25,26^, Alexander Huege MD^24^, Fiona Leonard MD^9^, Ian K Maconochie MD PhD^27,28^, Robin Marlow MD PhD^29^, Gregorio Milani MD PhD^3,4^, Miguel Angel Molina Gutierrez MD PhD^30^, Anna Maria Musolino MD^31^, Rianne Oostenbrink MD PhD^32^, Zanda Pucuka MD^33^, Simon Rivière MD^25^, Damian Roland Md PhD^34,35^, Malin Ryd Rinder MD PhD^36^, Maria Chiara Supino MD^31^, Ozlem Teksam MD PhD^12^, Victoria Trenchs MD PhD^19^, Zaba Valtuile Liebert PhD^2^, Bento Vanda MD^14^, Sanne Vrijlandt MD^32^, Naim Ouldali MD PhD^2,37^, Ruud G Nijman MD PhD^28,38^ , Elena Bellelli MD^31^, Ron Berant MD PhD^39^, Liviana Da Dalt MD PhD^15^, Patrick Fitzpatrick MD^40^, Florian Hey MD^41^, Florian Hoffman MD^41^, Lina Jankauskeite MD PhD^42^, Mark Lyttle MD^29^, Patricia Macao MD^43^, Lia Mano MD^18^, Henriette A Moll MD PhD^32^, Rosa Morello MD^16^, Sofia Reis MD^44^, Francisca Saraiva MD^14^, Keren Shahar MD PhD^39^, Roberto Velasco MD PhD^45^, Anna Chiara Vittucci MD^31^, Zsolt Bognar MD PhD^46^

**Affiliations**

^1^ Pediatric Emergency Department, Robert Debré University Hospital, Assistance Publique–Hôpitaux de Paris, Paris Cité University, Paris, France

^2^ Université Paris Cité and Université Sorbonne Paris Nord, Inserm, IAME, F-75018, France

^3^ Pediatric Emergency Department, Forndazione IRCCS Ca’ Granda Ospedale Maggiore Policlinico, Milan, Italy

^4^ Department of Clinical Science and Community Health, University of Milan, Milan, Italy

^5^ Centro Hospitalar e Universitário de São João, university of Porto

^6^ Pediatric Emergency Department, Jean Verdier Hospital, Bondy, Assistance Publique-Hôpitaux de Paris, Sorbonne Francecité, France

^7^ INSERM UMR 1123, ECEVE, Paris, France

^8^ Pediatric Emergeny Department, Ondokuz Mayıs University, Samsun, Turkey

^9^ Pediatric Emergency Department, Children’s Health Ireland at Crumlin, Dublin, Ireland

^10^ Women’s and Children’s Health, University College Dublin, Dublin, Ireland

^11^ Pediatric Emergency Department, Louis Mourier Hospital, Colombes, Assistance Publique–Hôpitaux de Paris, Paris Cité University, Paris, France

^12^ Division of Pediatric Emergency Medicine, Department of Pediatrics, Hacettepe University School of Medicine, Ankara, Turkey

^13^ Department of Pediatrics, NoordWest Ziekenhuisgroep, Alkmaar, The Netherlands

^14^ Departamento da Criança e do Jovem- Urgencia Pediatrica, Hospital Prof. Doutor Fernando da Fonseca, Amadora, Portugal

^15^ Division of Pediatric Emergency Medicine, Department of Women’s and Children’s Health – University Hospital of Padova, Italy

^16^ Department of Woman and Child Health and Public Health, Fondazione Policlinico Universitario Agostino Gemelli IRCCS, 00168 Rome, Italy.

^17^ Area Pediatrica, Dipartimento di Scienze della Vita e di Sanità Pubblica, Università Cattolica del Sacro Cuore, 00168 Rome, Italy.

^18^ Pulmonology Unit, Department of Pediatrics, Hospital Dona Estefania, Centro Hospitalar Lisboa Central, Portugal.

^19^ Pediatric Emergency Department, Hospital Joan de Déu Barcelona. University of Barcelona. Environment Effects on Child/Adolescent Well-being, Institut de Recerca Sant Joan de Déu. Barcelona, Spain

^20^ Department of Pediatric Emergency Medicine, Children’s Health Ireland at Tallaght, Ireland

^21^ Discipline of Paediatric Medicine, Trinity College Dublin, Dublin, Ireland

^22^ Pediatric Emergency Department, Szent Gyorgy University Teaching Hospital of Fejer County, Szekesfehervar, Hungary

^23^ Biobizkaia Health Research Institute, Paediatric Emergency Department, Hospital Universitario Cruces, Barakaldo, Basque Country, Spain.

^24^ Pediatric Emergency Unit, Clinical Division of Pediatric Pulmonology, Allergology and Endocrinology, Department of Pediatrics and Adolescent Medicine, Medical University of Vienna, Vienna, Austria

^25^ Pediatric Emergency Department, Armand Trousseau Hospital, Assistance Publique–Hôpitaux de Paris, Sorbonne Université,

^26^ CRESS Inserm U-1153 Paris, Epopé Team, Paris , France

^27^ Department of Pediatric Emergency Medicine, Division of Medicine, St. Mary’s hospital; Imperial College NHS Healthcare Trust, London, UK

^28^ Center for Pediatrics and Child Health, Faculty of Medicine, Imperial College London, UK

^29^ Emergency Department, Bristol Royal Hospital for Children, Bristol, UK; Faculty of Health and Applied Sciences, University of the West of England, Bristol, UK

^30^ Pediatric Emergency Department, Hospital Universitario La Paz, Madrid, Spain

^31^ Emergency Department, Bambino Gesù Children’s Hospital, IRCCS, Rome, Italy

^32^ Department General Pediatrics, ErasmusMC – Sophia, Rotterdam, The Netherlands

^33^ Paediatric emergency department, Children’s Clinical University Hospital, Riga Stradins University, Riga, Latvia

^34^ SAPPHIRE Group, Population Health Sciences, Leicester University, Leicester, UK

^35^ Pediatric Emergency Medicine Leicester Academic (PEMLA) Group, Leicester Hospitals, Leicester, UK

^36^ Pediatric emergency department, Astrid Lindgrens Children’s hospital, Karolinska University Hospital, Sweden

^37^ Department of General Pediatrics, Pediatric Infectious Disease and Internal Medicine, Robert Debré University Hospital, Assistance Publique-Hôpitaux de Paris, Paris, France; Paris Cité University

^38^ Section of Paediatric Infectious Diseases, Department of Infectious Diseases, Imperial College London

^39^ Pediatric Emergency Department, Schneider Children’s Medical Center of Israel And Sackler faculty of medicine

^40^ Pediatric Emergency Department, Children’s Health Ireland at Temple Street, Ireland

^41^ Pediatric Intensive Care Unit and Emergency Department, Dr. von Hauner Children’s Hospital, Ludwig-Maximilians-University Munich, Munich, Germany

^42^ Lithuanian University of Health Sciences, Department of Pediatrics; Kaunas, Lithuania

^43^ Pediatric Emergency Service, Hospital Pediátrico, Centro Hospitalar e Universitário de Coimbra, Portugal

^44^ Pediatric Department, Centro Hospitalar Tondela-Viseu, Viseu, Portugal

^45^ Pediatric emergency unit, Hospital Universitario Río Hortega, Valladolid, Spain

^46^ Pediatric Emergency Department, Heim Pal National Pediatric Institute, Budapest, Hungary
